# Postprandial Glycogen Content Is Increased in the Hepatocytes of Human and Rat Cirrhotic Liver

**DOI:** 10.3390/cells10050976

**Published:** 2021-04-21

**Authors:** Natalia N. Bezborodkina, Sergey V. Okovityi, Boris N. Kudryavtsev

**Affiliations:** 1 Zoological Institute, Russian Academy of Sciences, Universitetskaya nab. 1, 199034 St. Petersburg, Russia; 2 Department of Pharmacology and Clinical Pharmacology, Saint Petersburg State Chemical Pharmaceutical University, 197022 St. Petersburg, Russia; okovityy@mail.ru; 3 Scientific-Clinical Centre, Pavlov First Saint Petersburg State Medical University, L’va Tolstogo str. 6-8, 197022 St. Petersburg, Russia; bn_kudryavtsev@mail.ru

**Keywords:** liver cirrhosis, glycogen, hepatocytes, glycogen phosphorylase, glycogen synthase, glucose-6-phosphatase

## Abstract

Chronic hepatitises of various etiologies are widespread liver diseases in humans. Their final stage, liver cirrhosis (LC), is considered to be one of the main causes of hepatocellular carcinoma (HCC). About 80–90% of all HCC cases develop in LC patients, which suggests that cirrhotic conditions play a crucial role in the process of hepatocarcinogenesis. Carbohydrate metabolism in LC undergoes profound disturbances characterized by altered glycogen metabolism. Unfortunately, data on the glycogen content in LC are few and contradictory. In this study, the material was obtained from liver biopsies of patients with LC of viral and alcohol etiology and from the liver tissue of rats with CCl_4_-induced LC. The activity of glycogen phosphorylase (GP), glycogen synthase (GS), and glucose-6-phosphatase (G6Pase) was investigated in human and rat liver tissue by biochemical methods. Total glycogen and its labile and stable fractions were measured in isolated individual hepatocytes, using the cytofluorometry technique of PAS reaction in situ. The development of LC in human and rat liver was accompanied by an increase in fibrous tissue (20- and 8.8-fold), an increase in the dry mass of hepatocytes (by 25.6% and 23.7%), and a decrease in the number of hepatocytes (by 50% and 28%), respectively. The rearrangement of the liver parenchyma was combined with changes in glycogen metabolism. The present study showed a significant increase in the glycogen content in the hepatocytes of the human and the rat cirrhotic liver, by 255% and 210%, respectively. An increased glycogen content in cells of the cirrhotic liver can be explained by a decrease in glycogenolysis due to a decreased activity of G6Pase and GP.

## 1. Introduction

Chronic hepatitises of various etiologies are widespread, dangerous liver diseases in humans [1]. Their final stage, liver cirrhosis (LC), results in liver failure and significantly increases the risk of primary carcinoma and cholangiocarcinoma, as well as malignant tumors in other organs [2,3,4]. The development of LC is accompanied by a dramatic restructuring of the parenchyma, a significant expansion of its cicatricial tissue and a decrease in the number of hepatocytes [5,6,7]. A progressively decreasing number of hepatocytes and the deterioration of the conditions of their functioning under conditions of cirrhosis result in an impairment of the numerous tissue-specific functions of the liver. One of the most important of these functions is the ability of the liver to store large amounts of glycogen and to break it down according to the body’s needs.

Glycogen is a readily available reservoir of energy for diverse metabolic processes in many mammalian organs and tissues. Its content in the liver is a sensitive indicator not only of the state of energy reserves in the cells but also of carbohydrate metabolism in the body. Relatively few data on the glycogen content in the cirrhotic liver are currently available. The studies of glycogen content in human and rat liver have shown that it is much lower in cirrhotic liver as compared to normal liver [8,9,10,11]. At the same time, the data obtained in these studies do not allow one to make an unambiguous conclusion about the glycogen content and its metabolism in cirrhotic liver because they employed indirect methods, and their results heavily depend on the accuracy of the determination of fibrous tissue volume and hepatocyte fraction in the examined material.

The aim of this work was to determine the activity of the key enzymes of glycogen metabolism and its content in human and rat liver biochemically and, importantly, with the use of the cytofluorimetric method, which makes it possible to measure glycogen directly in the hepatocytes regardless of the fibrous tissue volume in the liver.

## 2. Materials and Methods

### 2.1. Patients, Animals, and Procedures

*Patients.* The first group consisted of 13 patients with liver cirrhosis of mixed viral and alcoholic etiology (males, mean duration of the disease 6.8 ± 1.3 years). All the patients (1) had HCV antibodies and a detectable HCV RNA viral load; (2) were in the “at-risk” drinking group (alcohol consumption for men of an age of up to 65 years, on average, of 14 or more drinks per week or 4 or more drinks per occasion AUDIT-C > 16 [12]); (3) did not consume any alcohol in the past 30 days. The second group served as controls and consisted of 10 volunteers whose livers were revealed by histological, immunochemical, and biochemical methods to have no lesions (males).

Supravital punctate liver biopsies were obtained from patients and volunteers at the same time (from 10 a.m. to 11 a.m.) on an empty stomach, 14 h after eating a meal. The biopsy samples were used for histological, biochemical, and cytophotometric research. All participants from both groups underwent a biochemical blood test.

This study was reviewed and approved by the Ethics Committee St. Petersburg State Medical Academy named after I.I. Mechnikov, St. Petersburg, Russian Federation, and was performed in accordance with the Declaration of Helsinki (1989) of the World Medical Association (IRB number: 707/60). All the patients and volunteers gave written informed consent for participation in the study, including the publication of its results.

*Animals.* We used 10 outbred adult male white rats with a body weight of about 140 g at the beginning of the experiment. The rats were obtained from Rappolovo breeding farm (Leningrad region) and were fed a complete feed ration (Laboratorkorm, Russia; Russian GOST 2874-82) and water ad libitum.

The rats were randomized into an experimental group and a control group, five rats in each. The rats from the experimental group inhaled CCl_4_ vapours three times weekly for 20 min (7 mL per 100 l of the closed chamber volume) for six months. Rats from the control group were untreated. A week after the termination of treatment with CCl_4_ rats from both groups were pre-anesthetized with hexobarbital sodium (60 mg/kg) and then sacrificed by decapitation after nocturnal fasting. The fasting time was 14 h. Their blood samples and pieces of liver were used for histological, biochemical, and cytophotometric research.

The experiments comply with the ARRIVE guidelines (https://www.nc3rs.org.uk/arrive-guidelines (accessed on 12 March 2021)) and were carried out in accordance with European Communities Council Directive of 24 November 1986 (86/609/EEC) (https://op.europa.eu/en/publication-detail/-/publication/cc3a8ccb-5a30-4b6e-8da8-b13348caeb0c/language-en (accessed on 12 March 2021)) and the European Community legislation (2010/63/UE) with the approval of the Ethics Committee of Saint Petersburg Chemical Pharmaceutical Academy. 

### 2.2. Tissue Preparation

Pieces of rat liver (~4 mm^3^) and human liver biopsies (~2 mm^3^) were fixed in 10% neutral formaldehyde for at least 48 h at 20–22 °C and embedded in paraffin blocks. Histological sections, approximately 5 µm thick, were obtained with the help of the Reichert microtome (Wien, Austria). To reveal the connective tissue, the sections were stained with picro-fuchsin after Van Gieson (Biovitrum, Russia) or with Picrosirius red (0.01% solution of sirius red F3BA (Bio-Optica Milano SPA, Milan, Italy).

### 2.3. Histological Analysis

A percentage of the connective tissue and the parenchyma of the rat and human liver in the sections were assessed by a VideoTest image analyzer with a 10×/0.30 objective and light interference filter of λmax = 500 nm. For each animal and man, 20–30 fields of vision were analyzed. The proportion of connective tissue in the liver was calculated using the following formula: Q = Sct/(Sav − Svb), where Q—the proportion of connective tissue from the cut area; Sav—the area of the field of view of the microscope (μm^2^); Sct—connective tissue area (μm^2^); Svr—the area of the lumens of blood vessel and tissue breaking (μm^2^).

The number of hepatocytes in the liver was determined using the formula

N = P × R × f/M

where N—number of hepatocytes in the liver; P—raw weight of the liver, g; R—the proportion of the parenchyma in the liver; f—coefficient of conversion from raw liver weight to dry; M is the average dry weight of one hepatocyte, g. The coefficient f was taken to be equal to 0.27 [13].

### 2.4. Smears of Isolated Hepatocytes on Object Slides

A piece of rat liver (~2 mm^3^) and human biopsies (~1 mm^3^) were placed in phosphate buffer I, pH = 8.0 (475 mL 0.066 M Na_2_HPO_4_·2H_2_O, 25 mL 0.067 M KH_2_PO_4_, 500 mL 0.15 M sucrose), for 10 min and then in phosphate buffer II, pH = 7.4 (400 mL Na_2_HPO_4_·2H_2_O, 100 mL KH_2_PO_4_), for 15 min. The piece was gently stirred in a drop of buffer II with the help of pincers in order to obtain a suspension of cells, which was smeared on an object slide using quartz glass with a polished edge. Immediately after that, the smears of the isolated hepatocytes were treated twice with 100% methanol by carefully dropping them from a pipette at 20–22 °C, and they were left to dry for about 3–5 min. The smears were then stored in the dark [14].

### 2.5. Determination of Dry Weight of Hepatocytes (DWH)

DWH was measured with the help of an interference microscope MBIN-4 (LOMO, St. Petersburg, Russia, http://lomoplc.com/, accessed on 21 April 2021). We equipped this microscope with a CCD-camera connected to a computer with a morphometric software program “VideoTesT-Morphometry” (Ista-Videotest Ltd., St. Petersburg, Russia, http://www.videotest.ru/ (accessed on 12 March 2021)). DWH was calculated according to the formula [15]:

$$P=\frac{\delta \cdot S}{100\cdot \alpha }$$

where *P*—dry weight of the cell (in pg), *δ*—path-length difference (in cm). *δ* was determined according to the formula *δ* = (φ1 − φ2)∙λ/K, where φ1 and φ2—readings of the Senarmont compensator scale, λ—length of the light wave (546 nm), K = 180°, S—cell area (in cm^2^), *α*—specific increment of the refractive index, making up 0.00095 cm^3^/g for the proteins in glycerine [16]. 

### 2.6. Determination of Total Glycogen Content (TG) and Its Labile (LF) and Stable (SF) Fractions in Hepatocytes

The preparations were stained for glycogen with a fluorescent variant of the Periodic acid–Schiff (PAS) reaction, with auramine-SO_2_ (Au-SO_2_) used as Schiff’s reagent. Smears of isolated hepatocytes were placed in a potassium periodate solution (200 mg potassium periodate, 25 mL 0.23% HNO_3_) for 1.5 h. They were then washed under running water for 5 min, rinsed once in distilled water, and placed into a 0.3% SO_2_-saturated auramine solution (0.2 mL thionyl chloride per 100 mL of the dye) for 40 min (for LF determination) or 90 min (for TG determination). After that, the preparations were taken out and rinsed thrice in fresh distilled water and thrice in sulfur waters (5 g K_2_S_2_O_5_, 950 mL water, 50 mL 1N HCl) for 3 min in each case, washed under running water for 20 min, rinsed in distilled water, and placed into an ascending alcohol series (70°, 96°, and 100°) for 5 min in each alcohol. Before measurements, the stained preparations were embedded into a non-fluorescent paraffin oil. The SF was calculated as the difference between TG and LF.

**Figure 1:**
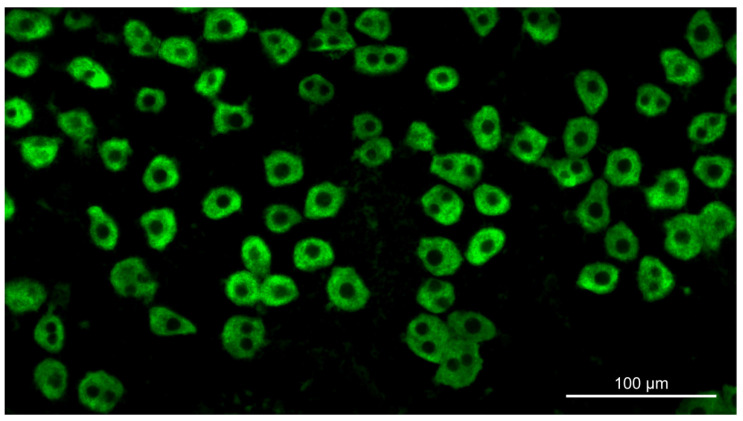
Glycogen detection in hepatocytes by fluorescent PAS reaction.

Fluorescent images of Au-SO_2_-stained hepatocytes (Figure 1) were obtained with the use of an Axioskop microscope (Carl Zeiss, Jena, Germany, http://www.zeiss.com/ (accessed on 12 March 2021)) equipped with a Plan-NEOFLUAR 20×/0.50 objective, Filter Set 10, and a digital high-sensitivity CCD camera Leica DFC420C (Leica Microsystems, Wetzlar, Germany, http://www.leica-microsystems.com/ (accessed on 12 March 2021)). Fluorescence intensity of 100–150 cells obtained from each animal or from each human was measured with the use of the ImageJ software (National Institutes of Health, Bethesda, MD, USA, http://rsb.info.nih.gov/ij/ (accessed on 12 March 2021)).

### 2.7. The Blood Serum Analysis

Following blood sample collection into plain tubes, samples were centrifuged at 3500 rpm for 10 min at +4 °C. Using automatic biochemical analyzers SMA-12/16 (Technicon Instruments Co., Mequon, WI, USA) and Abbott-Spectrum (Abbott Laboratories, Lake Forest, IL, USA), the activity of alanine aminotransferase (ALT), aspartate aminotransferase (AST), and alkaline phosphatase (ALP), as well as the concentration of total bilirubin (TB), total protein (TP), and glucose (Glu) were determined in the rat and human blood serum. 

### 2.8. Glycogen Concentration in Rat Livers

Liver tissues were lysed in 30% KOH in a boiling water bath for 60 min, centrifuged for 30 min at 1000 g, washed in an ascending ethanol series (70, 80, and 96%), and then centrifuged again. The glycogen pellet was hydrolyzed in 2N H_2_SO_4_ in a boiling water bath for 2.5 h. The hydrolysate was neutralized with 5N NaOH up to pH 7.8–8.0, and the amount of the glucose formed was determined by the glucose oxidase method.

### 2.9. GS and GP Activity in Rat and Human Livers

Livers were homogenized in 50 mM Tris-HCl buffer (pH 7.4) with 5 mM EDTA, 200 mM sucrose, 0.01M β-mercaptoethanol, and 0.2 M PMSF on ice (1:10). The homogenate was centrifuged at 1000× *g* and 4 °C for 10 min to remove incompletely destroyed cells and nuclei. The supernatant was then centrifuged at 14,000× *g* and 4 °C for 10 min and used to determine the activity of GS and GP by the substrate-labelled assay.

GS activity was estimated by the amount of [U-^14^C]glucose included in the glycogen, using UDP-[U-^14^C]glucose (300 mCi/mM) as a substrate; GP activity was determined using the inverse reaction of glycogen synthesis in vitro using [U-^14^C]glucose-1-phosphate (286 mCi/mM) as a substrate [17,18]. The number of impulses was registered with a counter (Beckman, Indianapolis, IN, USA).

### 2.10. G-6-Pase Activity in Rat and Human Livers

The activity of G-6-Pase was determined in the resuspended microsome pellet [19] using [U-^14^C]-glucose-6-phosphate (49 mCi/mM, Sigma-Aldrish, St. Louis, MO, USA) as a substrate. For this, 80 μL of the reaction mixture (pH 6.5) containing 50 mM cacodylic acid, 2 mM EDTA, 10 mM glucose-6-phosphate (30,000 cpm), and 20 μL of microsomal suspension were incubated for 20 min at 30 °C. After incubation, to separate the labeled glucose-6-phosphate that did not react during the reaction, 50 μL of the mixture was applied to a 1.2 cm column prepared from a Pasteur pipette with Dowex-1 anion exchange resin (×8, in acetate form), which was then washed with two portions of water 0.5 mL each. The effluents were then collected into vials and used for counting impulses.

### 2.11. Protein Content in Rat and Human Livers

The enzyme activity depended on the protein content in the sample and was proportional to the incubation time. The protein content was identified following Bradford [20], with the use of Coomassie brilliant blue G-250. Protein content was read from the standard calibration curve, which was constructed from measurements of various amounts of BSA at 595 nm with the use of the Specol 11 spectrophotometer (Carl Zeiss, Jena, Germany).

### 2.12. Statistical Analysis

Statistical treatment of the results was performed using Sigma Plot for Windows 11.0 standard software package (Systat Software Inc., Chicago, IL, USA). Normality of distribution of quantitative variables was tested using the Shapiro–Wilk W-test. The data were given as mean ± standard error of the mean. Differences between the mean values were detected using Student’s t-criterion.

## 3. Results

The body weight of the cirrhotic rats decreased, while the absolute and the relative liver weight increased as compared to those of the control group (Table 1). Cirrhosis in rats resulted in pronounced changes in the biochemical parameters of the blood serum, which indicated a significant weakening of protein synthesis, impaired bilirubin metabolism, and a considerable rate of hepatocyte death. ALT and AST levels increased 2.0- and 1.4-fold, respectively, while the concentration of total bilirubin in blood increased 1.7-fold. The concentration of the total protein in the blood of rats with LC decreased by 5.6% (*p* < 0.05). At the same time, the concentration of glucose in the blood of rats with LC did not differ from the normal values (Table 1).

Control and cirrhotic patients are characterized in Table 2. Age and body weights were not different between cirrhotic and control patients. All patients were in the postabsorptive state but not starving. Metabolic characterization revealed no differences in glucose serum concentrations between cirrhotic and control patients. Cirrhotic patients had decreased protein serum concentrations as well as higher serum levels of bilirubin. The activities of alanine aminotransferase, aspartate aminotransferase, and alkaline phosphatase were increased in patients with liver cirrhosis.

ALT, AST, ALP, and total bilirubin levels in LC patients were, respectively, 4.7, 2.5, 1.7, and 2.2 times higher than the normal ones, while serum total protein concentration decreased by 9.2% (*p* < 0.0001) (Table 2). At the same time, the glucose level in the blood of cirrhotic patients did not differ from the normal levels.

**Figure 2:**
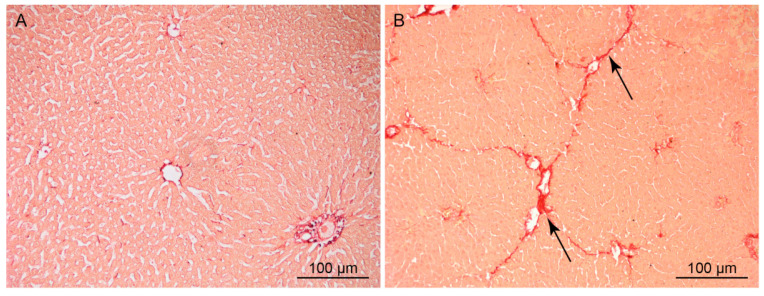
Normal (**A**) and cirrhotic (**B**) rat liver sections. Picrosirius red stain; arrows indicate connective tissue.

Chronic intoxication of rats by CCl_4_ for 26 weeks induced LC, which was characterized by a significant increase in the fibrous tissue volume in the parenchyma and the death of the hepatocytes (Figure 2A,B). The proportion of cicatricial tissue increased 8.8-fold in the rat liver during LC development, and the number of hepatocytes in the cirrhotic rat liver decreased by ~28% as compared to the norm. The dry weight of the hepatocyte in the cirrhotic liver increased as compared to normal liver, on the average by 25.6% (Table 3).

**Figure 3:**
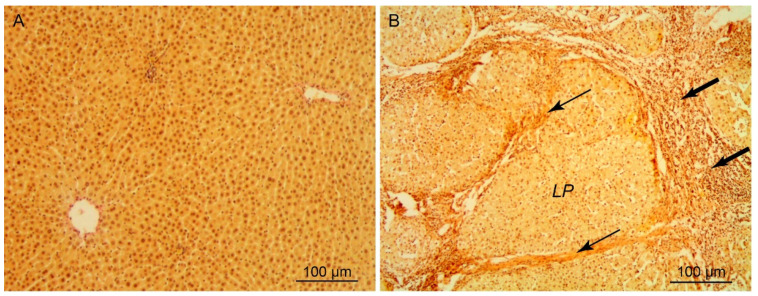
Normal (**A**) and cirrhotic (**B**) human liver sections. Haematoxylin-picrofuchsin stain; *LP*—liver pseudolobule; bold arrows indicate necrotic foci; thin arrows indicate of connective tissue.

The morphometric parameters of the human cirrhotic liver are shown in Table 4. As cirrhosis develops, the proportion of parenchyma in the human liver decreased in comparison with the norm and made up 54%. Correspondingly, the proportion of cicatricial tissue in the course of the pathological process increased approximately by a factor of 20, from 1.8% to 36.5%. A dramatic rearrangement of the liver parenchyma structure in the case of cirrhosis (Figure 3A,B) was accompanied by a high rate of death of hepatocytes, whose number decreased in comparison with normal liver by more than 50%. The death of hepatocytes stimulated a regenerative response of the liver, which was expressed in the hypertrophy of the hepatocytes and the increase in their ploidy. As a result, the dry weight of hepatocytes in the cirrhotic liver increased, on the average by 23.7%, partially compensating for the loss of these cells.

**Figure 4:**
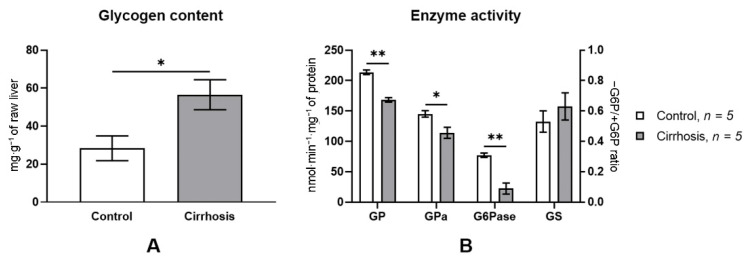
Liver glycogen content (**A**) and glycogen metabolism enzyme activity (**B**) in normal and cirrhotic rat liver. GP—total glycogen phosphorylase; GP*a*—glycogen phosphorylase *a*; G6Pase—glucose-6-phosphatase; * *p* < 0.05; ** *p* < 0.0001. GS activity was measured as the activity ratio in the absence of G6P relative to that in the presence of G6P. Glycogen content and enzyme activities were normalized for fibrotic tissue content.

A dramatic rearrangement of the lobular structure and a considerable increase in the volume of the fibrous tissue in the liver of cirrhotic rats brought about pronounced changes in the activity of the glycogen metabolism enzymes. Total GP activity and GPa activity in the cirrhotic liver decreased almost equally, by 21% (*p* < 0.0001), as compared to the norm. G6Pase activity in the cirrhotic liver decreased by 71.1% (*p* < 0.0001), while GS activity was unchanged. The glycogen content in the liver of cirrhotic rats almost doubled as compared to the normal liver (*p* < 0.05) (Figure 4).

**Figure 5:**
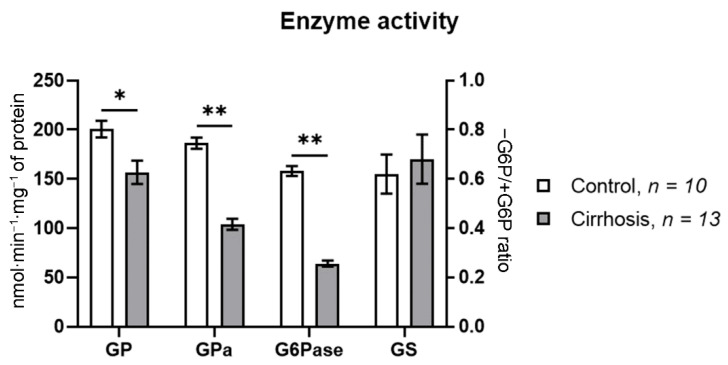
Glycogen metabolism enzyme activity in normal and cirrhotic human liver. GP—total glycogen phosphorylase; GP*a*—glycogen phosphorylase *a*; G6Pase—glucose-6-phosphatase; *—cirrhosis vs. control at *p* < 0.001; **—cirrhosis vs. control at *p* < 0.0001. GS activity was expressed as the ratio of its activity in the absence of G6P to that in the presence of G6P. Enzyme activities were normalized for fibrotic tissue content.

The development of cirrhosis brought about important changes in the activity of key enzymes of glycogen metabolism in the human liver. The changes in the activity of glycogen phosphorylase and glucose-6-phosphatase were particularly prominent. The activity of total phosphorylase in the cirrhotic liver decreased by 21.9% as compared to the normal liver (*p* < 0.001), while the activity of its active form, GPa, decreased by 44.2% (*p* < 0.0001). In the normal human liver, the proportion of GPa in total glycogen phosphorylase activity made up 92.9%, while in the cirrhotic liver it fell to 66.4%. The activity of glucose-6-phosphatase in the cirrhotic liver decreased even more significantly, by 59.5% compared to the norm. The activity of GS in the cirrhotic liver did not differ from that in the normal one (Figure 5).

**Figure 6:**
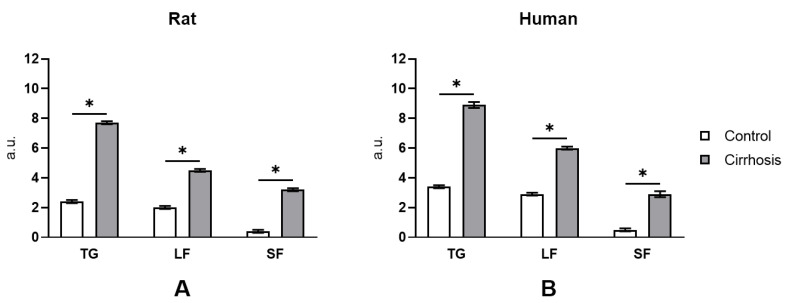
Cytofluorimetric study of total glycogen content and its fractions in normal and cirrhotic rat (**A**) and human (**B**) hepatocytes. A.u.—arbitrary units; TG—total glycogen; LF—labile fraction; SF—stabile fraction; *—*p* < 0.0001.

Direct determination of glycogen content in hepatocytes of the normal and the cirrhotic liver of humans and rats using cytofluorimetric PAS reaction (Figure 6) showed that the glycogen content was considerably higher in the cirrhotic liver. Glycogen content per hepatocyte increased on the average by 210% in the cirrhotic human liver as compared to the normal human liver and by ~255% in the cirrhotic rat liver as compared to the normal rat liver. The accumulation of glycogen in the hepatocytes of the cirrhotic liver was accompanied by a change of the proportion of its fractions indicating a transformation of the glycogen structure. In the hepatocytes of the normal human and rat liver, the acid-labile fraction (LF) of glycogen predominated, making up 83–85%. The acid-stable fraction (SF), which can be completely extracted from the cells only after treatment with strong alkali, made up 14.7% and 16.7% in the normal human and the normal rat liver, respectively. Cirrhosis is characterized by a significant change in the composition of the glycogen fractions: the proportion of LF decreases, while that of SF increases considerably (Figure 6).

## 4. Discussion

Chronic exposure of animals to CCl_4_ inhalation is a traditional way of inducing experimental LC [21]. Combined exposure to CCl_4_ and phenobarbital is often used to accelerate LC [9,22,23]. However, phenobarbital itself has been shown to affect the glycogen content in the liver [24], to modulate the activity of the enzymes of its metabolism [25], and to increase liver weight [26]. Therefore, in order to induce LC in rats, we exposed them to CCl_4_ only.

Repeated exposure to a hepatotoxin causes important changes in the structure of the rat liver, which have been described in detail previously [27,28]. A characteristic change in the cirrhotic liver is a considerable increase in the connective tissue. Bands of connective tissue of varying thickness pass across the parenchyma, dividing it into so-called “false lobules”, often lacking central veins. Necrotic areas, extensive infiltrates of leucocytes, and numerous Kupffer cells can be seen in the parenchyma. As a result, the original lobular structure of the organ undergoes a profound rearrangement (Figure 2), which leads to an impairment of the liver blood vessels and has severe consequences for the liver function. In the LC model used in our study, the proportion of the connective tissue in the liver increased 8.8-fold after exposure to CCl_4_. The rearrangement of the parenchyma results in a lowered supply of oxygen and nutrients to the hepatocytes and, as a consequence, to their death. The results presented in the table indicate that the number of hepatocytes in the cirrhotic rat liver decreased by 28.2% in comparison with the control animals. Continuous death of hepatocytes and the impairment of numerous liver functions stimulate regenerative processes, whose main cellular mechanisms are proliferation, polyploidization, and cell hypertrophy [13,29]. Increased ploidy and hypertrophy of hepatocytes leads to an increase in their weight, which increases in the cirrhotic liver by 25.6% on average (Table 3). Thus, the increase in hepatocyte weight almost entirely compensates for the loss of these cells during the development of cirrhosis in rats.

Compared with experimental liver cirrhosis in rats, human cirrhotic liver is characterized by a more profound rearrangement of the parenchyma (Figure 3). The differences are manifested not only in a greater volume of the connective tissue in the cirrhotic human liver (36.5% compared to 7.3% in cirrhotic rat liver), but also in a greater extent and thickness of connective-tissue bands. In addition, the cirrhotic human liver is characterized by a higher loss of hepatocytes, 51% as compared with 28% in the cirrhotic rat liver. Although during cirrhosis development in the human liver the hepatocyte weight does increase by 23.7%, owing to the regenerative process this increase does not compensate for the loss of the cells (Table 4). A greater severity of LC in humans also manifests itself in more marked changes in blood plasma parameters, which characterize the state of the liver (Table 1 and Table 2). ALT activity in the blood serum of humans with LC increased 4.7-fold (in rats with LC, 1.9-fold) as compared to the norm; AST activity increased 2.5-fold (in rats, 1.4-fold); and bilirubin concentration increased 2.2-fold (in rats, 1.7-fold). The concentration of total protein in the serum during LC in humans decreased by 9.8% (in rats, by 5.6%), as compared to the norm.

It may be assumed that a fundamental factor behind the differences in the severity of LC in rats and humans is the difference in their metabolic rate, which is in turn due to the difference in the body weight. A much lower metabolic rate in humans [30] prolongs the impact of the harmful factor in the body and results in more severe liver damage as compared to rats.

Although glucose levels in the blood of cirrhotic rats and humans in the postprandial period do not differ from the norm (Table 1 and Table 2), the metabolism of glucose in the case of LC undergoes significant changes [8,31,32,33]. An important indicator of the state of glucose metabolism in the liver is the content of glycogen, the level of which depends on the rates of its synthesis and degradation. The activity of GS and GP plays a key role in these processes.

According to biochemical analysis, in the postprandial period the concentration of glycogen in the cirrhotic liver of rats doubled as compared to the norm (56.5 ± 7.9 mg/g raw liver weight vs. 28.3 ± 6.5 mg/g, *p* < 0.05) (Figure 4A). This result disagrees with the earlier data obtained from other LC models. In particular, combined exposure to CCl_4_ and phenobarbital has been shown to reduce postprandial glycogen content by ~36% per hepatocyte. Since the activity of GS and GP in the cirrhotic liver did not differ from the control values, the authors concluded that a decreased glycogen content in LC was due to intrinsic causes [9]. Using the model of biliary LC in rats, glycogen content per milliliter of hepatocytes was found to fall by ~61% [10]. The authors explain the decrease in glycogen reserves by a decrease in the rate of its synthesis due to low GS activity. Finally, using thioacetamide LC model, it was shown that 4 h after the start of refeeding the glycogen stores in the cirrhotic rat liver were only half of those of the control animals. A slower replenishment of the glycogen reserves in the cirrhotic rats as compared to the control was also attributed to a low GS activity [34].

A possible reason behind the difference between our data and those of other authors may be a different technique of determination of the volume of hepatocytes in the cirrhotic liver, whose accuracy to a large extent determines that of the calculated glycogen content in the liver. To exclude the influence of this factor, we measured the glycogen content using the cytofluorimetric method. When this method is used, the volume of hepatocytes in the cirrhotic liver can be ignored, because the glycogen content is measured directly in the hepatocytes. Cytofluorometry of glycogen in rat hepatocytes showed that glycogen content in the cells of the cirrhotic liver was much higher than that in normal ones (Figure 6A), which confirms the results of the biochemical analysis (Figure 4A).

The determination of the activity of the key enzymes of glycogenesis and glycogenolysis in the rat liver showed that the levels of both total GP and its active form, GPa, were reduced by about 20% in the cirrhotic liver (Figure 4A). The activity of GS in the cirrhotic rat liver did not differ from the control values. These data confirm the results of our previous studies [14,35] and suggest that the glycogen accumulation in hepatocytes may be associated with a decreased activity of the key enzyme of glycogenolysis, GPa.

Several studies are known in which the glycogen content has been determined in the cirrhotic human liver. However, their results are ambiguous. The study of biopsied material showed that glycogen content made up 25.8 ± 3.5 mg/g raw weight in patients with alcoholic cirrhosis, while in two healthy individuals it made up 32.0 and 48.8 mg/g raw weight [8]. These values approximately correspond to an average glycogen content, 43.7 ± 1.8 mg/g, in the liver of 58 healthy volunteers after 12–16 h of overnight fasting [36]. Owen and co-authors attributed the decreased glycogen content in the cirrhotic liver to the considerable rearrangement of the liver architecture as well as an increased fibrosis: the proportion of cicatricial tissue in the liver parenchyma of the cirrhotic patients was ~41 ± 3% [8]. Since in the normal human liver the parenchyma occupies about 90% of its volume [37], the data of Owen et al. suggest that the fibrosis-free parenchyma of patients with alcoholic cirrhosis makes up 49% of the liver and glycogen concentration in it is 52.6 ± 7.1 mg/g (25.8 ± 3.5 mg/g/0.49 = 52.6 ± 7.1 mg/g), which is not different from the glycogen concentration in the normal human liver, 43.7 ± 1.8 mg/g [36].

In another study using biopsied material from the liver of patients with alcoholic and biliary cirrhosis, the glycogen content per milliliter of hepatocytes was found to be ~50% lower than in the normal liver [11]. At the same time, GS activity as well as GP and GPa activity did not differ between patients in the control group and the LC group. The authors attributed such a significant decrease in glycogen content in the liver of patients with cirrhosis to a sharp decrease in glucokinase (GK) activity, but the mechanism of this decrease remained unclear.

Finally, in a study employing the ^2^H_2_O method and ^13^C nuclear magnetic resonance spectroscopy (NMR), it was shown that glycogen concentration in the liver of cirrhotic patients was 34% lower than in the control group. Unfortunately, glycogen concentration in this work was calculated against the total cirrhotic liver volume rather than the volume of the parenchyma, and the decrease in the glycogen content is not evident. It is also surprising that the 34% reduction in glycogen content in the liver of cirrhotic patients was accompanied by a 3.5-fold decrease in glycogenolysis [38].

Glycogen cytofluorometry in hepatocytes of the normal and the cirrhotic human liver showed that the glycogen content in parenchyma cells of the cirrhotic liver was more than twice as high as in the normal one (Figure 6B). Glycogen accumulation in human cirrhotic hepatocytes was accompanied by a marked decrease in GP and GPa activity, by 22% and by 46%, respectively (Figure 6B). This result confirms our earlier data [14,18,35]. We conclude that the abatement of glycogenolysis due to a low activity of GPa may be an important mechanism of increasing glycogen concentration in the cirrhotic human liver. An equally significant factor of glycogen metabolism in the cirrhotic liver is a significant decrease in G6Pase activity: 3.46-fold in rats and 2.47-fold in humans (Figure 5B and Figure 6B), which fully conforms to our earlier findings [14,35].

A considerable rearrangement of the lobular structure of the liver during the formation of LC leads to a decreased ATP production by mitochondria, cell death, and the development of hypoxia [39,40,41,42]. Hypoxia accompanies chronic liver impairment of any etiology and increases mitochondrial production of reactive oxygen species (ROS) [40,41,43,44]. Increased production of ROS after the failure of antioxidant mechanisms is a common characteristic of a sustained inflammatory response to liver impairment. It causes the formation of active lipid peroxidation products, such as lipid hydroperoxides, diene conjugates, malondialdehyde, etc., that alter the properties of cell membranes. Since G6Pase is a microsomal enzyme embedded into the membranes of the endoplasmic reticulum (ER) [45], many believe that the disruption of the structure of ER membranes due to the damaging effects of lipid peroxidation products is one of the main causes of a reduced glucose-6-phosphatase system activity in the cirrhotic liver [46,47].

G6Pase is a key enzyme of glucose production in the liver. It catalyzes the terminal reactions of gluconeogenesis and glycogenolysis, which are among the major suppliers of glucose-6-phosphate (G6P) in hepatocytes. The concentration of G6P in hepatocytes regulates the rate of synthesis (glycogenesis) and breakdown (glycogenolysis) of glycogen [48]. Under physiological conditions, G6P concentration is inversely correlated with GPa activity; therefore, metabolic conditions promoting an increase in G6P concentration in hepatocytes inhibit GPa and so slow down the rate of glycogenolysis [49]. Thus, it can be assumed that a significant decrease in G6Pase activity in the cirrhotic liver contributes to an increase in the G6P concentration in hepatocytes, an increase in GS activity, and glycogen synthesis and, as a consequence, promotes its accumulation in cirrhotic liver cells.

## 5. Conclusions

The present study showed a more than two-fold increase in the glycogen content in the hepatocytes of the cirrhotic liver in humans and rats. An increase in the content of glycogen in the cells of the cirrhotic liver can be explained by a decrease in glycogenolysis due to a decrease in the activities of glucose-6-phosphatase and glycogen phosphorylase.

## Figures and Tables

**Table 1:** Characterization of the animals. Data are given as mean ± SE; *n*—number of animals in each group; ALT—alanine aminotransferase; AST—aspartate aminotransferase.

Parameter	Control, *n* = 5	Cirrhosis, *n* = 5
Body weight, g	324 ± 13	267 ± 19 ^1^
Liver weight, g	9.72 ± 0.37	11.56 ± 0.55 ^1^
Relative liver weight, %	3.00 ± 0.06	4.33 ± 0.09 ^3^
Serum glucose, mmol/l	5.54 ± 0.18	5.47 ± 0.09
Plasma ALT, mmol/l	143.2 ± 5.2	278.7 ± 9.1 ^3^
Plasma AST, mmol/l	160.8 ± 8.1	228.0 ± 14.6 ^2^
Plasma bilirubin, µmol/l	0.34 ± 0.04	0.58 ± 0.04 ^2^
Plasma total protein, g/l	6.46 ± 0.14	6.10 ± 0.03 ^1^

^1^*p* < 0.05 vs. control; ^2^ *p* < 0.01 vs. control; ^3^ *p* < 0.001 vs. control.

**Table 2:** Characterization of the humans. Data are given as mean ± SE; *n*—number of patients in each group; ALT—alanine aminotransferase; AST—aspartate aminotransferase.

Parameter	Control, *n* = 10	Cirrhosis, *n* = 13
Age, y	44.7 ± 5.0	50.3 ± 4.7
Body weight, g	71.2 ± 3.5	67.4 ± 4.2
Serum glucose, mmol/l	4.64 ± 0.28	4.76 ± 0.19
Plasma ALT, mmol/l	0.31 ± 0.02	1.46 ± 0.20 ^1^
Plasma AST, mmol/l	0.34 ± 0.02	0.84 ± 0.07 ^1^
Plasma bilirubin, µmol/l	14.50 ± 0.13	31.9 ± 6.2 ^1^
Plasma total protein, g/l	85.7 ± 1.3	77.8 ± 1.1 ^1^
Alkaline phosphatase, mmol/l	98.38 ± 7.25	169.78 ± 4.36 ^1^

^1^*p* < 0.0001 vs. control.

**Table 3:** Morphometric parameters of the normal and the cirrhotic rat liver. Data are given as mean ± SE; *n*—number of animals in each group.

Parameter	Control, *n* = 5	Cirrhosis, *n* = 5
Proportion of parenchyma, %	88.0 ± 1.1	79.2 ± 1.3 ^2^
Proportion of connective tissue, %	0.84 ± 0.07	7.31 ± 0.27 ^2^
Average dry weight of hepatocyte, pg	943 ± 21	1184 ± 56 ^2^
Number of hepatocytes/g liver wet weight	(2.52 ± 0.15) · 10^8^	(1.81 ± 0.21) · 10^8 1^

^1^*p* < 0.05 vs. control; ^2^ *p* < 0.001 vs. control.

**Table 4:** Morphometric parameters of the normal and the cirrhotic human liver. Data are given as mean ± SE; *n*—number of patients in each group.

Parameter	Control, *n* = 10	Cirrhosis, *n* = 13
Proportion of parenchyma, %	90.6 ± 0.9	54.0 ± 2.5 ^2^
Proportion of connective tissue, %	1.79 ± 0.08	36.5 ± 3.3 ^2^
Average dry weight of hepatocytes, pg	546.0 ± 29.6	675.4 ± 41.3 ^1^
Number of hepatocytes/g liver wet weight	(4.81 ± 0.27) · 10^8^	(2.32 ± 0.29) · 10^8 2^

^1^*p* < 0.05 vs. control; ^2^ *p* < 0.0001 vs. control.

## Data Availability

Not applicable.
